# Core Symptoms Between Adolescent Psychological Abuse-Neglect and Impulsiveness: A Network Analysis

**DOI:** 10.31083/AP44085

**Published:** 2025-06-20

**Authors:** Yuhang Wu, Yuqin Song, Lu Pan, Cen Lin, Yu Cen, Mengqin Dai, Qiuyue Fan, Jiarui Shao, Cailin Xie, Jiaming Luo

**Affiliations:** ^1^Mental Health Center, Affiliated Hospital of North Sichuan Medical College, 637000 Nanchong, Sichuan, China; ^2^School of Psychiatry, North Sichuan Medical College, 637000 Nanchong, Sichuan, China

**Keywords:** adolescent behavior, impulsiveness, mental health, network analysis, psychological abuse, psychological neglect

## Abstract

**Background:**

Psychological abuse and neglect are considered fundamental to the development of impulsiveness. However, the interplay between psychological abuse-neglect symptoms and impulsiveness dimensions remains unclear, especially during adolescence, a critical developmental stage. This study uses network analysis to explore the link between adolescent psychological abuse-neglect and impulsivity, aiming to inform targeted early interventions and treatment strategies for impulsive behaviors.

**Methods:**

Cluster sampling was used to gather demographic data from 6731 students across 17 middle schools. Clinical assessments utilized the Chinese Barratt Impulsiveness Scale (BIS-11) and Child Psychological Abuse and Neglect Scale (CPANS). Network analysis explored associations between the six CPANS components and three impulsiveness dimensions. Centrality indices and stability indicators were calculated.

**Results:**

In the study population, 47.4% were female, and 68.4% were middle school students. Scolding (4.0 [1.0, 7.0]) scored highest in abuse, while Emotional Neglect (6.0 [2.0, 12.0]) scored highest in neglect. Among impulsive types, Non-planning Impulsiveness (47.5 [32.5, 60.0]) ranked highest. Emotional Neglect emerged as the central node in the network, with the greatest strength, closeness, and influence, while Non-planning Impulsiveness showed the highest correlation with centrality. All centrality indices had Correlation Stability (CS) coefficients of 0.75, with narrow 95% confidence intervals for edge weights.

**Conclusions:**

This study underscores the central role of emotional neglect in the development of impulsive traits in adolescents. Network analysis revealed that emotional neglect serves as a critical link between abuse-neglect and impulsivity, with non-planning impulsivity acting as a key mediator. The results emphasize the need for comprehensive interventions, as well as addressing the impact of early traumatic experiences.

**Clinical Trial Registration:**

The study was registered at https://www.chictr.org.cn/showproj.html?proj=134138, registration number: ChiCTR2100052297, date of registration: 24 October 2021.

## Main Points

1. Network analysis revealed that emotional neglect holds a central position in the network structure of adolescent psychological abuse and impulsivity and is the most influential node.

2. Non-planning acts as a bridging node, linking psychological abuse-neglect to other impulsive behaviors, highlighting its significant role in the network structure.

3. This study suggests that interventions should prioritize the regulation of emotional neglect and impulsivity traits to mitigate their long-term negative impact on adolescent mental health.

4. The findings support a strong association between emotional neglect and impulsivity traits, emphasizing the potential value of early interventions targeting emotional neglect to reduce impulsive behaviors among adolescents.

## 1. Introduction

The World Health Organization defines child abuse and neglect as any form of physical, emotional, or sexual abuse, negligence, or exploitation that results in actual or potential harm to a child’s health, development, or dignity within a relationship of responsibility, trust, or power [1]. There are four types of child abuse—sexual abuse, physical abuse, emotional abuse, and neglect [2]. Child abuse has become a global public health issue. In China, the study reveal increasing rates of abuse among primary and secondary students: 20% for physical abuse, 30% for emotional abuse, 12% for sexual abuse, 47% for physical neglect, and 44% for emotional neglect [3]. Child abuse is linked to a range of negative outcomes, including violent behavior, emotional disorders, self-injury, and substance use. It often leads to early onset of clinical symptoms, more severe disease progression, and poorer treatment outcomes [4]. The long-term effects of abuse can persist into adulthood, contributing to mental disorders and suicide. Additionally, child abuse has profound social and economic consequences, including increased disability, healthcare costs, and social inequality [5]. Understanding child abuse is crucial due to its widespread impact on individual health and society.

Compared to other forms of abuse and neglect, psychological abuse and neglect in children are more difficult to identify and address [6, 7]. Child psychological abuse and neglect (CPAN) refers to ongoing and repeated inappropriate parenting practices. Specifically, abuse involves the use of language and expressions by parents to threaten or humiliate children, restrict them, or encourage inappropriate behaviors, without involving physical or sexual contact. Neglect refers to the prolonged failure of parents to meet their children’s needs [8]. In traditional Chinese culture, parents often view psychological abuse of children (such as harsh scolding) as expressions of love and care. They tend to employ stricter, hostile, rejecting, or neglectful behaviors in child education. This makes psychological abuse and neglect a potentially significant public health issue in China [9]. Previous research suggests that child abuse and neglect affect psychological well-being through behavioral, emotional, and cognitive pathways. These effects often persist into adolescence, a critical stage for psychological development, and have profound impacts on personality traits, including impulsiveness [10].

Impulsivity refers to the tendency to act quickly without considering the potential negative consequences. It is generally categorized into three dimensions: non-planning, motor, and cognitive impulsiveness [11]. Research by Felitti *et al*. (1998) [12] has highlighted a significant association between childhood experiences of psychological abuse and neglect and the development of impulsive traits. Cumulative adverse experiences, such as psychological abuse and neglect, disrupt emotional regulation and increase the likelihood of impulsive behaviors during both adolescence and adulthood [13]. These experiences affect the brain’s emotional and behavioral control systems, resulting in long-term changes in impulsivity. Impulsivity also serves as a mediator between psychological abuse and neglect and negative psychological outcomes, such as depression, anxiety, and substance abuse [14]. Studies suggest that impulsiveness, driven by psychological abuse and neglect, contributes to the development of various psychological and behavioral disorders. A deeper understanding of impulsivity can provide valuable insights into the biological and neurological mechanisms underlying these disorders. Integrating impulsivity into research can improve intervention strategies aimed at mitigating the effects of psychological abuse and neglect. By recognizing impulsivity as a key mediating factor, we can develop more effective treatments for individuals affected by early trauma [15].

The diathesis-stress model provides a useful framework for understanding how psychological abuse and neglect contribute to impulsivity [16]. According to this model, individuals with an inherent predisposition for impulsivity (diathesis) are more vulnerable to exhibiting impulsive behaviors when exposed to external stressors such as neglect or abuse. Research consistently shows a significant association between childhood trauma and increased impulsivity in adulthood [17]. For instance, Liu [18] (in the meta-analysis) found a positive correlation between trait impulsivity and childhood abuse, with a stronger link in cases of emotional abuse. These findings suggest that individuals who experience emotional, physical, or sexual abuse often struggle with emotional regulation. As a result, they may rely on impulsive behaviors as coping mechanisms to manage emotional stress and negative emotions [19]. To explore these complex dynamics, this study utilized network analysis, a method that effectively captures the intricate interactions between the symptoms of abuse, neglect, and impulsivity. Fried *et al*. (2017) [20] outlined the use of network analysis in psychological research, particularly for understanding the interactions between symptoms. This approach identifies central symptoms within the network and elucidates their interactions, offering valuable insights into how psychological abuse contributes to impulsive behaviors.

Although existing evidence suggests an association between childhood psychological abuse and neglect and impulsivity traits, it remains unclear which specific symptomatic characteristics of psychological abuse and neglect are most closely related to impulsivity traits, and to what extent common factors explain the relationship between these two phenomena. Therefore, we hypothesize that psychological abuse and neglect may be related to impulsivity traits, and that specific impulsivity traits may have different associations with the characteristics of psychological abuse and neglect. This study employs network analysis to clarify the relationships between these dimensions and to provide empirical support for clinical interventions.

## 2. Methods and Procedure

### 2.1 Data Collection Methods, Inclusion and Exclusion Criteria

This study was conducted between November 2021 and May 2022 using a cluster random sampling method, with questionnaires distributed to 17 randomly selected middle schools in Sichuan Province. The inclusion criteria were as follows: students aged 10 to 19 years who are currently enrolled in middle or high school, with no history of serious physical illnesses, and who have agreed to participate in the study and are able to complete the questionnaire. Exclusion criteria included adolescents with psychiatric disorders, which were assessed using the Kiddie Schedule for Affective Disorders and Schizophrenia (K-SADS), as well as individuals with intellectual disabilities, any serious physical illness, a history of suicide attempts, or those who had taken antidepressants, mood stabilizers, or antipsychotic medications within the past week. All assessments were conducted by licensed psychiatrists.

The research team consisted of faculty members and graduate students from the Department of Mental Health at the North Sichuan Medical College. Prior to the survey, all participants were trained in relevant professional knowledge and questionnaire content. The training included the study background, relevant research topics, field survey methods, and procedures. A standard operating procedure (SOP) was developed to ensure that assessors followed a uniform standard for explaining, distributing, collecting, and reviewing questionnaires, as well as managing and auditing the entire process for handling the questionnaires. Throughout the data collection process, all researchers strictly adhered to standardized procedures, distributing electronic questionnaires to participants. The students completed the surveys.

This study was a cross-sectional study. This cross-sectional study employed bilateral testing with a significance level of α = 0.05 and a tolerance error of δ = 0.01. According to the literature, the estimated incidence rate of impulsivity traits in adolescents is 20%. Using PASS15 software (version 15, developed by NCSS, LLC, Kaysville, UT, USA, https://www.ncss.com), the minimum required sample size was calculated to be 6250 participants. To account for a 10% potential data loss or refusal rate, the sample size was adjusted to 6945 participants. After removing cases with missing data, clearly inconsistent answers, fabricated responses, and extreme outliers—defined as scores more than three standard deviations above the mean—the final sample size consisted of 6731 adolescents.

### 2.2 Measures

#### 2.2.1 Demographic Variables

A comprehensive survey was conducted using a custom-designed questionnaire developed by the investigator, covering various parameters including gender (male or female), educational level (middle or high school), and other relevant sociodemographic factors.

#### 2.2.2 Psychological Abuse and Neglect

Psychological abuse and neglect were assessed using the Children’s Psychological Abuse and Neglect Scale (CPANS) [21], a self-reported measure designed to evaluate the extent of psychological abuse and neglect experienced by children and adolescents. The CPANS consists of 31 items, divided into two subscales: psychological abuse and psychological neglect. The psychological abuse subscale includes items related to verbal abuse, emotional manipulation, and threats, while the psychological neglect subscale assesses the lack of emotional support, neglectful supervision, and failure to meet emotional needs.

Data collection was conducted through structured interviews and self-administered questionnaires, where participants were asked to rate the frequency of each experience on a 5-point Likert scale: 0 = never, 1 = rarely, 2 = sometimes, 3 = often, and 4 = always. Higher total scores indicate a greater severity of psychological abuse and neglect during childhood. The purpose of obtaining these data was to quantify the impact of psychological abuse and neglect on adolescents, focusing on their potential influence on mental health, behavior, and emotional regulation. By examining the frequency and severity of these experiences, we sought to identify adolescents at risk of developing maladaptive behaviors such as impulsivity. In this sample, Cronbach’s alpha was 0.941, indicating excellent internal consistency and reliability of the scale.

#### 2.2.3 Impulsiveness

Impulsivity traits in participants were assessed using the Chinese version of the Barratt Impulsiveness Scale (BIS-11) [22], which includes three subscales: non-planning impulsiveness, motor impulsiveness, and cognitive impulsiveness. The BIS-11 is a self-reported questionnaire designed to evaluate different dimensions of impulsivity. For data collection, participants were instructed to respond to a series of statements related to their typical behaviors and thought patterns, using a 4-point Likert scale ranging from “rarely/never” to “almost always”. Some items were reverse-scored to ensure consistency and prevent response biases, with higher total scores indicating higher levels of impulsivity. The purpose of using the BIS-11 was to quantitatively measure impulsivity traits in adolescents and explore their potential relationship with psychological abuse and neglect. Understanding the extent of impulsivity in relation to these psychosocial factors may help identify adolescents at greater risk of engaging in impulsive or maladaptive behaviors, thereby assisting in developing preventive strategies and interventions. The scale demonstrated good internal consistency, with a Cronbach’s alpha of 0.80, indicating reliable measurements.

### 2.3 Statistical Analysis

We utilized SPSS Statistics 26.0 (IBM Corporation, Armonk, NY, USA) to perform descriptive statistical analyses, providing insights into the sample’s distribution characteristics. To assess gender differences in psychological abuse, neglect, and impulsive traits, the non-parametric Mann-Whitney U test was conducted, comparing male and female participants’ scores on these variables.

Network analysis is a statistical technique that uses a graphical representation to illustrate the relationships between variables and the strength of their correlations. Unlike traditional methods that rely on predefined dimensions, network analysis reveals the complex, interconnected nature of symptoms without such restrictions [23]. This approach allows for a more intuitive understanding of how symptoms interact and identifies which symptoms are central within the network. For this study, network analysis was applied to explore the relationships between psychological abuse, neglect, and impulsivity.

Network analysis was performed using the q graph package (version 1.9.2) in R software (version 4.4.1, R Core Team, R Foundation for Statistical Computing, Vienna, Austria, https://www.r-project.org/), constructing the network using Gaussian Graphical Models (GGMs). GGMs were first used to estimate partial correlation coefficients between nodes. Given the large number of nodes (e.g., 9 nodes require estimating 36 parameters: 9 threshold parameters and 27 pairwise correlation parameters), this can lead to false-positive edges. To address this, we employed the graphical lasso (glasso) algorithm for regularization, producing a sparse inverse covariance matrix where many elements are zero, indicating conditional independence between corresponding variables. The regularization process of the glasso algorithm was combined with the Extended Bayesian Information Criterion (EBIC) to optimize model selection. EBIC introduces a penalty term to control model complexity and reduce the risk of overfitting. The estimate Network function automatically implements the glasso regularization.

We used the q graph package to plot the network graph. In the network graph, nodes represent individual variables, and edges indicate the relationships between variables [24]. To better visualize the data, we grouped the nodes and explored different layout options, including the spring layout and circle layout. Spring Layout: This is a force-directed layout algorithm that arranges nodes on a 2D plane by simulating attractive and repulsive forces between them, bringing connected nodes closer together and pushing unconnected nodes further apart. Circle Layout: In this layout, all nodes are positioned around a circle, with nodes from each group (or community) placed in separate circles to facilitate the visualization of relationships between different communities and the overall structure.

We measured centrality indices of the established network, including node strength, closeness centrality, and betweenness centrality. Node strength refers to the sum of the absolute values of the edge weights connected to a node. Closeness centrality reflects the average distance from one node to all other nodes in the network. Betweenness centrality indicates the number of times a node appears on the shortest path between other nodes. To assess the stability of the centrality indices, we conducted analyses using network model estimation based on data subsets and case-dropping bootstrapping (N = 1000). If the values dropped significantly after removing participants, the centrality indices were considered unstable. The robustness of the network was evaluated using the bootstrapping method in the R package bootnet. The stability analysis was calculated using the CS coefficient (Correlation Stability Coefficient), which represents the maximum proportion of cases that can be dropped while retaining a correlation greater than 0.7 (default) for the original centrality at a 95% confidence level.

## 3.Results

### 3.1 Demographic Characteristics

This study surveyed a total of 6731 adolescents, of whom 47.4% were female and 68.4% were middle school students. We performed a non-parametric Mann-Whitney U test to compare male and female participants’ scores on psychological abuse-neglect and impulsivity traits. The results showed that the relationship between psychological abuse-neglect and impulsivity was not significantly different in gender (*p* > 0.05). Scolding was the highest-rated category of abuse, with a score of (4.0 [1.0, 7.0]), followed by Intimidation (3.0 [1.0, 6.0]) and Interfering (2.0 [0.0, 6.0]). Among the reported neglect categories, Emotional Neglect had the highest score at (6.0 [2.0, 12.0]), followed by Educational Neglect (2.0 [0.0, 5.0]) and Physical Neglect (2.0 [0.0, 5.0]). Regarding impulsivity traits, Non-planning Impulsiveness had the highest score at (47.5 [32.5, 60.0]), followed by Cognitive Impulsiveness (47.5 [32.5, 57.5]) and Motor Impulsiveness (30.0 [15.0, 45.0]). The median, interquartile spacing among the main study variables are shown in Table 1.

**Table 1.** **Descriptive characteristics of the sample**.

Variables		M (P_25_, P_75_) or n (%)
Gender	Male	52.6%
	Female	47.4%
Educational level	Junior high school	68.4%
	Senior high school	31.6%
Abuse	Scolding	4.0 (1.0, 7.0)
	Intimidation	3.0 (1.0, 6.0)
	Interference	2.0 (0.0, 6.0)
Neglect	Emotional neglect	6.0 (2.0, 12.0)
	Education neglect	2.0 (0.0, 5.0)
	Physical neglect	2.0 (0.0, 5.0)
Impulsiveness	Non-planning Impulsiveness	47.5 (32.5, 60.0)
	Motor Impulsiveness	30.0 (15.0, 45.0)
	Cognitive Impulsiveness	47.5 (32.5, 57.5)

### 3.2 Network Structure

**Fig. 1.**
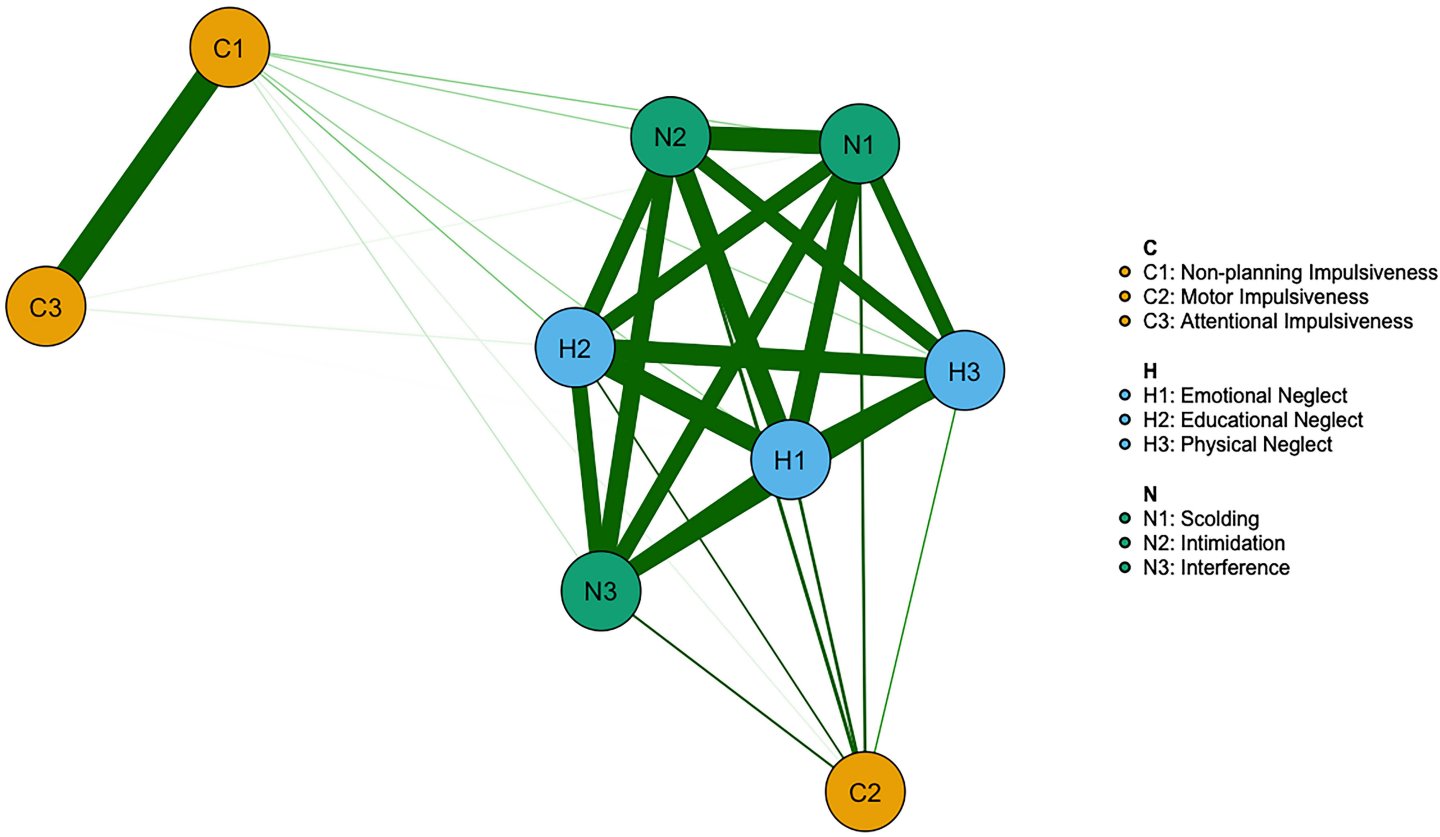
**Psychological network of abuse-neglect and impulsiveness**.

The network of impulsiveness and psychological abuse-neglect is shown in Fig. 1. Highly correlated nodes are positioned close to each other, while weakly correlated nodes are farther apart. Different-colored nodes in the figure represent various types of impulsiveness, psychological abuse, and neglect, with varying connection strengths between node types, indicating their relative independence in the network. The connections within specific groups are strong, and no negative edges were found. Among the 36 edges, all weights were greater than zero. For example, the strongest edge in the impulsiveness (weight = 0.87) was between Non-planning Impulsiveness and Cognitive Impulsiveness. In the psychological neglect symptoms, the strongest edge (weight = 0.81) was between Emotional Neglect and Educational Neglect. For psychological abuse, the strongest edge (weight = 0.87) was between Scolding and Intimidation. Notably, there was a strong connection (weight = 0.80) between Physical Neglect in the psychological neglect group and Interfering in the abuse group. Here, the weights represent the regularized regression coefficients.

### 3.3 Centrality Indices

**Fig. 2.**
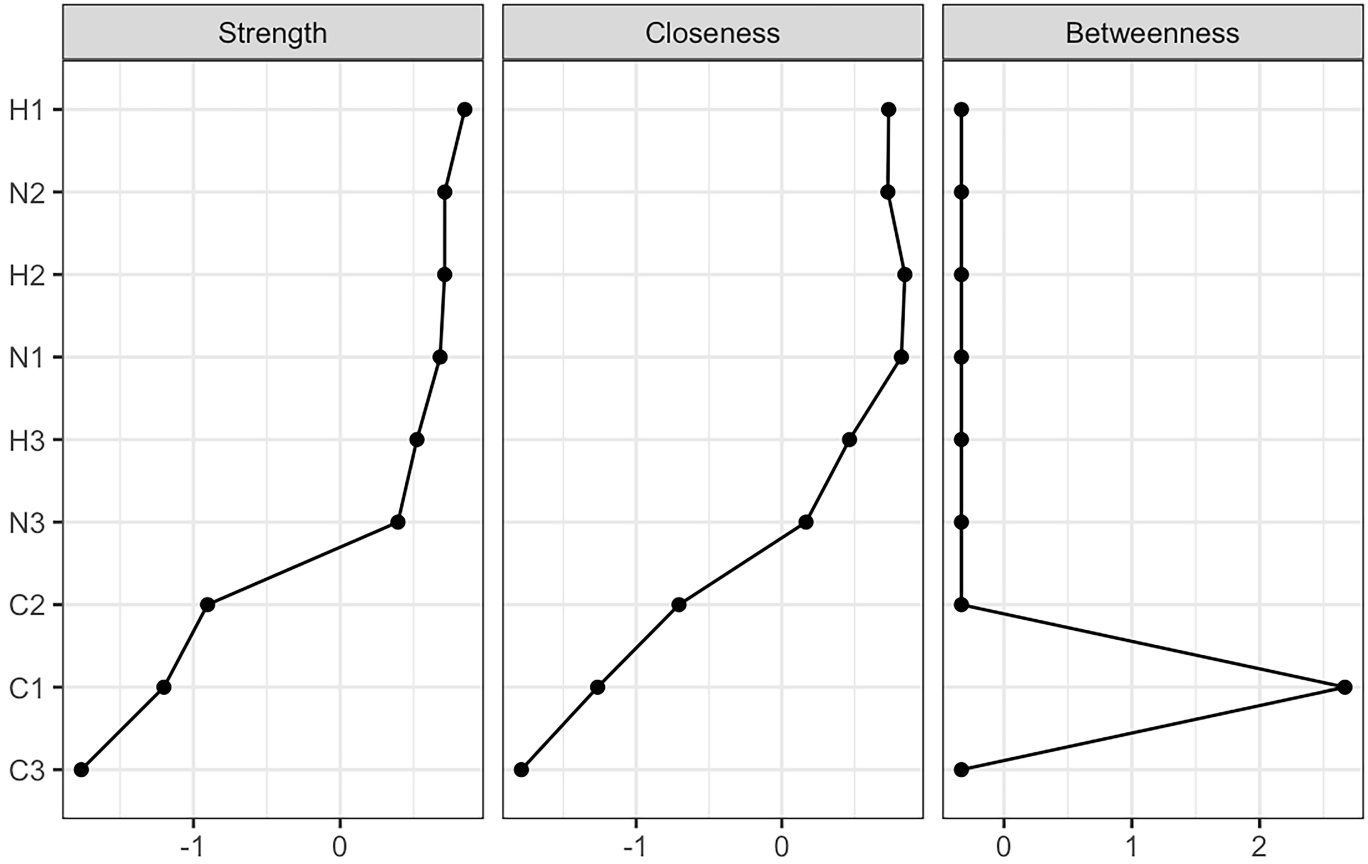
**Centrality indices of network nodes**. The nodes represent distinct factors: impulsivity (C1: Non-planning Impulsiveness, C2: Motor Impulsiveness, C3: Attentional Impulsiveness), neglect categories (H1: Emotional Neglect, H2: Educational Neglect, H3: Physical Neglect), and abuse categories (N1: Scolding, N2: Intimidation, N3: Interference).

Fig. 2 shows the centrality indices, with values scaled relative to the highest value for each measure (i.e., normalized). According to the four indices, Emotional Neglect (H1) from the neglect category emerged as the most important node symptom. In terms of strength, H1 (4.57) from the neglect category and Intimidation (N2: 4.43) from the abuse category ranked highest within their respective groups, indicating these nodes have the strongest relationships with other nodes and the greatest overall influence on the network. Next were N2 (4.429270) and Educational Neglect (H2: 4.428636). The lowest strength were seen in Attentional Impulsiveness (C3: 1.857121). In terms of betweenness and closeness, Non-planning Impulsiveness (C1: 7) from impulsiveness and H2 (0.055) from neglect symptoms ranked the highest, meaning they are closest to all other nodes in the network, with C1 playing the strongest bridging role within the network.

### 3.4 Stability of Centrality Indices

**Fig. 3.**
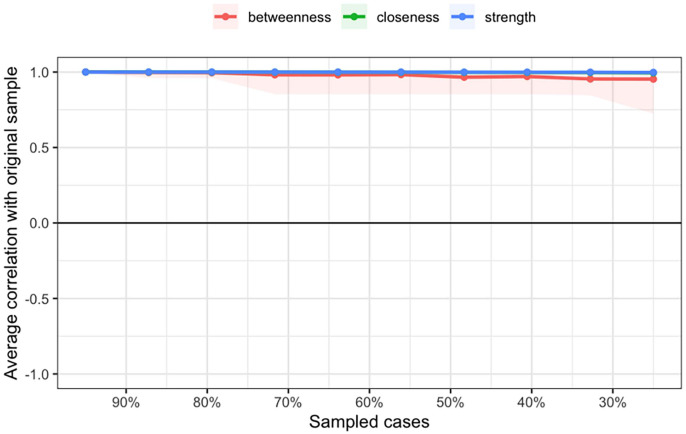
**Stability test of network centrality indices for adolescent psychological abuse-neglect and impulsivity traits**.

**Fig. 4.**
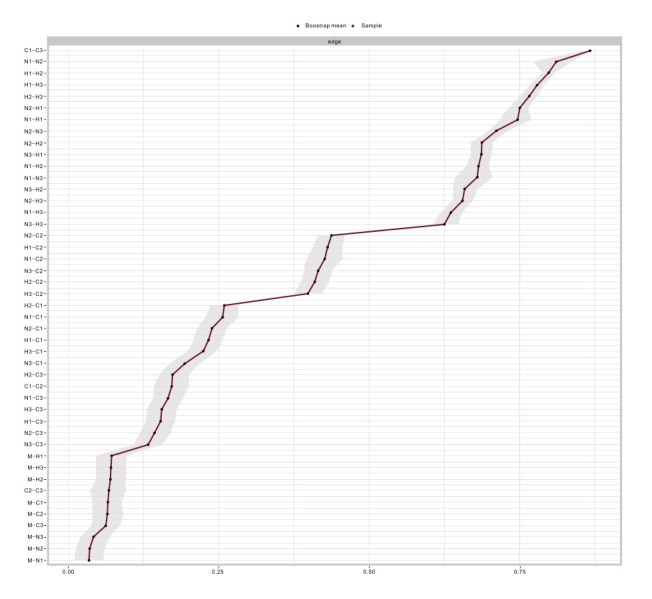
**Bootstrap confidence intervals for edges in the network**.

Fig. 3 shows the average correlations and confidence intervals of network centrality indices (Betweenness, Closeness, Strength) at different sample sizes, ranging from 100% to 30%. Each curve represents a centrality index and its correlation with the original sample at different sampling proportions. The CS coefficient is an important indicator for evaluating the stability of centrality indices. A CS coefficient of 0.75 indicates that even when the sample is reduced to 25% of the original size, these centrality indices still maintain a high correlation. All centrality indices have a CS coefficient of 0.75, indicating that they retain high correlations even when the sample size is reduced to 25%. This demonstrates that, regardless of sample size, the estimates of these centrality indices in the network analysis are stable and reliable. Fig. 4 shows that the bootstrap 95% confidence intervals of edge weights are relatively narrow, indicating sufficient accuracy. Among the different types of edge weights, the highest edge weights and shortest paths were found between Emotional Neglect and Intimidation, and between Emotional Neglect and Scolding.

## 4. Discussion

This study is the first to use network analysis to explore the association between impulsivity traits and different dimensions of psychological abuse and neglect among adolescents, providing empirical evidence to clarify the mechanism of their relationship. The results show a significant association between impulsivity traits in adolescents and their experiences of childhood abuse and neglect, with emotional neglect being the most prominent in the network structure. Our results further demonstrate how non-planning functions act as a mediator between emotional neglect and other forms of impulsive behaviors, offering new insights into the complex interplay between early adversity and impulsivity. This analysis is crucial for understanding the multifaceted nature of impulsivity and provides important implications for both theory and clinical practice.

The present study identifies emotional neglect as the central node in the network, exhibiting the highest intensity and expected impact among all nodes. Emotional neglect, characterized by the consistent failure to provide emotional support, affection, or responsiveness, significantly disrupts the development of effective emotion regulation mechanisms. Consistent with the emotional dysregulation theory, individuals who experience emotional neglect are more likely to struggle with managing their emotional responses, which often results in impulsive behavior [25]. From a neurobiological perspective, Braquehais *et al*. [17] found that neurobiological changes observed in individuals who had experienced childhood abuse and neglect were strongly correlated with neurobiological markers of impulsivity. Specifically, emotional neglect has been shown to disrupt the development of brain areas involved in impulse control, particularly the prefrontal cortex (PFC). PFC dysfunction may impair self-control and lead to increased impulsivity [26, 27]. This is consistent with our findings, where emotional neglect emerged as the most robust predictor of impulsivity in adolescents, emphasizing the importance of early emotional experiences in shaping long-term behavioral outcomes.

Additionally, emotional neglect not only directly influences other nodes but also indirectly affects the entire system through other nodes. The shorter path length further strengthens the connection between emotional neglect and the abuse nodes. This implies that emotional neglect may quickly escalate into more harmful behaviors, such as intimidation and scolding, thereby exacerbating the psychological distress of the victims [28]. This rapidly evolving chain of abusive behaviors suggests that emotional neglect is not only a distinct form of harm but also a potential catalyst for the development of other abusive behaviors [28, 29]. Therefore, emotional neglect may serve as the starting point for various psychological and behavioral issues, which underscores the need for more effective prevention and intervention measures to mitigate the negative impacts of these behaviors in children.

Non-planning impulsiveness has a significantly higher betweenness centrality than other nodes, indicating that it serves as a critical bridge in the network. Non-planning impulsiveness links neglect and abuse with other impulsive behaviors, making its position in the network particularly important. Evans *et al*. (2013) [30] pointed out that childhood experiences of abuse are significantly associated with impulsive behaviors in adulthood, and non-planning impulsiveness plays a critical role in transmitting the effects of emotional and physical abuse to other impulsiveness. Specifically, emotional neglect and abuse significantly increase negative emotions in children, and these negative effects are manifested through non-planning impulsiveness, making them less likely to exhibit foresight and planning when facing stress and challenges. As a result, they are more likely to engage in impulsive behaviors without considering the consequences. Research shows that individuals who lack effective strategies for coping with stress often resort to impulsive behaviors to alleviate inner unease and anxiety. This deficiency in coping strategies further reinforces the behavioral pattern of non-planning, making them more prone to impulsive and irrational actions when faced with new stressors. Non-planning impulsiveness also involves impairments in cognitive functioning. Existing research indicates that individuals who have experienced severe abuse exhibit significant deficits in executive functioning and decision-making abilities, which makes them more likely to display non-planning impulsiveness when faced with complex situations [30]. Additionally, in environments characterized by neglect and abuse, where there is a lack of positive behavioral guidance and support, children may develop a pattern of using impulsive behaviors to cope with their surroundings. This pattern is likely to become consistent in the absence of correction and guidance. Interventions targeting non-planning, such as Cognitive Behavioral Therapy (CBT), can help individuals improve their emotion regulation and impulse control abilities, thereby reducing the negative effects caused by childhood abuse and neglect [31]. Further research should also focus on how to effectively identify and treat these behavioral issues in order to reduce the occurrence of mental health problems during adolescence.

### Limitations

First, this study used a cross-sectional design to construct the network between impulsivity and psychological abuse-neglect, revealing significant associations. However, causal relationships cannot be determined from this design. Future research should use a longitudinal approach to clarify these causal links. Second, the pathways identified are based on specific measurement tools—namely, the Psychological Abuse and Neglect Scale (CPANS) and the Barratt Impulsiveness Scale (BIS-11). The findings may be influenced by the constructs measured by these scales, and the network structure could vary if a different theoretical framework, such as basic emotion science, were applied. Future studies should explore this variability by using alternative frameworks and tools. Lastly, the study sample was limited to a local population, which may not be representative of the broader population, potentially affecting the generalizability of the findings. Future research should aim for a more diverse and larger sample to enhance the robustness and reliability of the conclusions.

## 5. Conclusions

This study reveals how psychological neglect and abuse during childhood and adolescence are associated with impulsivity traits. The findings show that emotional neglect plays a central role not only among neglect symptoms but also within the relational network between impulsivity traits and abuse-neglect. Additionally, non-planning impulsivity acts as a bridge between neglect/abuse and impulsivity traits. These findings have several important implications for clinical practice. First, when assessing and intervening with children and adolescents who have experienced abuse or neglect, clinicians should pay particular attention to the presence of emotional neglect and its potential impact on impulsive behaviors. Second, interventions can focus on improving planning and decision-making abilities in children and adolescents, as early identification and treatment of these symptoms may help mitigate the long-term effects of impulsivity. Finally, these results highlight the importance of implementing comprehensive interventions in treatment, which should not only include emotional and behavioral regulation strategies, but also address the effects of early traumatic experiences.

By targeting these key bridging symptoms, interventions can help patients more readily access emotional regulation strategies, thereby maximizing treatment outcomes. Future research should continue to explore how different types of abuse and neglect affect impulsivity traits and identify other potential bridging symptoms. Longitudinal studies will help clarify the causal relationships of these symptoms, providing a solid scientific foundation for more effective prevention and intervention strategies.

## Data Availability

The authors will make the raw data supporting the conclusions of this article available upon request, without any undue restrictions.

## References

[1] World Health Organization (2022). Child Maltreatment. https://www.who.int/news-room/fact-sheets/detail/child-maltreatment.

[2] Norman RE, Byambaa M, De R, Butchart A, Scott J, Vos T (2012). The long-term health consequences of child physical abuse, emotional abuse, and neglect: a systematic review and meta-analysis. *PLoS Medicine*.

[3] Wang L, Cheng H, Qu Y, Zhang Y, Cui Q, Zou H (2020). The prevalence of child maltreatment among Chinese primary and middle school students: a systematic review and meta-analysis. *Social Psychiatry and Psychiatric Epidemiology*.

[4] Williams JMG, Crane C, Barnhofer T, Brennan K, Duggan DS, Fennell MJV (2014). Mindfulness-based cognitive therapy for preventing relapse in recurrent depression: a randomized dismantling trial. *Journal of Consulting and Clinical Psychology*.

[5] Fang X, Brown DS, Florence CS, Mercy JA (2012). The economic burden of child maltreatment in the United States and implications for prevention. *Child Abuse & Neglect*.

[6] Baker AJL, Brassard M (2019). Predictors of variation in sate reported rates of psychological maltreatment: A survey of statutes and a call for change. *Child Abuse & Neglect*.

[7] Baker AJL, Brassard MR, Rosenzweig J (2021). Psychological maltreatment: Definition and reporting barriers among American professionals in the field of child abuse. *Child Abuse & Neglect*.

[8] Liu F, Zhang Z, Chen L (2020). Mediating effect of neuroticism and negative coping style in relation to childhood psychological maltreatment and smartphone addiction among college students in China. *Child Abuse & Neglect*.

[9] Cui N, Xue J, Connolly CA, Liu J (2016). Does the gender of parent or child matter in child maltreatment in China?. *Child Abuse & Neglect*.

[10] Shin SH, Cook AK, Morris NA, McDougle R, Groves LP (2016). The different faces of impulsivity as links between childhood maltreatment and young adult crime. *Preventive Medicine*.

[11] Zhang J, Zhang X, Yang G, Feng Z (2022). Impulsiveness indirectly affects suicidal ideation through depression and simultaneously moderates the indirect effect: A moderated mediation path model. *Frontiers in Psychiatry*.

[12] Felitti VJ, Anda RF, Nordenberg D, Williamson DF, Spitz AM, Edwards V (1998). Relationship of childhood abuse and household dysfunction to many of the leading causes of death in adults. The adverse childhood experiences (ACE) study. *American Journal of Preventive Medicine*.

[13] Shorey RC, Brasfield H, Febres J, Stuart GL (2011). The association between impulsivity, trait anger, and the perpetration of intimate partner and general violence among women arrested for domestic violence. *Journal of Interpersonal Violence*.

[14] Hawes MT, Schwartz HA, Son Y, Klein DN (2023). Predicting adolescent depression and anxiety from multi-wave longitudinal data using machine learning. *Psychological Medicine*.

[15] Chen X, Li S (2023). Serial mediation of the relationship between impulsivity and suicidal ideation by depression and hopelessness in depressed patients. *BMC Public Health*.

[16] Nielsen JD, Mennies RJ, Olino TM (2020). Application of a diathesis-stress model to the interplay of cortical structural development and emerging depression in youth. *Clinical Psychology Review*.

[17] Braquehais MD, Oquendo MA, Baca-García E, Sher L (2010). Is impulsivity a link between childhood abuse and suicide?. *Comprehensive Psychiatry*.

[18] Liu RT (2019). Childhood Maltreatment and Impulsivity: A Meta-Analysis and Recommendations for Future Study. *Journal of Abnormal Child Psychology*.

[19] Lavi I, Katz LF, Ozer EJ, Gross JJ (2019). Emotion Reactivity and Regulation in Maltreated Children: A Meta-Analysis. *Child Development*.

[20] Fried EI, van Borkulo CD, Cramer AOJ, Boschloo L, Schoevers RA, Borsboom D (2017). Mental disorders as networks of problems: a review of recent insights. *Social Psychiatry and Psychiatric Epidemiology*.

[21] Pan C, Deng YL, Guan BQ, Luo XR (2010). Reliability and validity of child psychological maltreatment scale. *Chinese Journal of Clinical Psychology*.

[22] Yang HQ, Yao SQ, Zhu XZ, Auerbach RP, Abela JR, Tong X (2007). The Chinese version of the barratt impulsiveness scale, 11th version (BIS-11) in adolescents: Its reliability and validity. *Chinese Journal of Clinical Psychology*.

[23] Borsboom D, Cramer AOJ (2013). Network analysis: an integrative approach to the structure of psychopathology. *Annual Review of Clinical Psychology*.

[24] Epskamp S, Fried EI (2018). A tutorial on regularized partial correlation networks. *Psychological Methods*.

[25] Gross JJ (2015). Emotion regulation: Current status and future prospects. *Psychological Inquiry*.

[26] Teicher MH, Samson JA, Anderson CM, Ohashi K (2016). The effects of childhood maltreatment on brain structure, function and connectivity. *Nature Reviews*.

[27] Hanson JL, Nacewicz BM, Sutterer MJ, Cayo AA, Schaefer SM, Rudolph KD (2015). Behavioral problems after early life stress: contributions of the hippocampus and amygdala. *Biological Psychiatry*.

[28] Kumari V (2020). Emotional abuse and neglect: time to focus on prevention and mental health consequences. *The British Journal of Psychiatry: the Journal of Mental Science*.

[29] Gama CMF, Portugal LCL, Gonçalves RM, de Souza Junior S, Vilete LMP, Mendlowicz MV (2021). The invisible scars of emotional abuse: a common and highly harmful form of childhood maltreatment. *BMC Psychiatry*.

[30] Evans GW, Li D, Whipple SS (2013). Cumulative risk and child development. *Psychological Bulletin*.

[31] Loya JM, Benitez B, Kiluk BD (2023). The Effect of Cognitive Behavioral Therapy on Impulsivity in Addictive Disorders: a Narrative Review. *Current Addiction Reports*.

